# Gravitational-Wave Burst Signals Denoising Based on the Adaptive Modification of the Intersection of Confidence Intervals Rule

**DOI:** 10.3390/s20236920

**Published:** 2020-12-03

**Authors:** Nikola Lopac, Jonatan Lerga, Elena Cuoco

**Affiliations:** 1 Faculty of Maritime Studies, University of Rijeka, Studentska 2, 51000 Rijeka, Croatia; lopac@pfri.hr; 2 Center for Artificial Intelligence and Cybersecurity, University of Rijeka, Radmile Matejcic 2, 51000 Rijeka, Croatia; 3 Faculty of Engineering, University of Rijeka, Vukovarska 58, 51000 Rijeka, Croatia; 4 European Gravitational Observatory (EGO), Cascina, I-56021 Pisa, Italy; elena.cuoco@ego-gw.it; 5 Scuola Normale Superiore, Piazza dei Cavalieri, 7-56126 Pisa, Italy

**Keywords:** gravitational-waves, core collapse supernova (CCSN) signals, Advanced LIGO interferometers, adaptive signal denoising, local polynomial approximation (LPA), intersection of confidence intervals (ICI) rule, relative intersection of confidence intervals (RICI) rule

## Abstract

Gravitational-wave data (discovered first in 2015 by the Advanced LIGO interferometers and awarded by the Nobel Prize in 2017) are characterized by non-Gaussian and non-stationary noise. The ever-increasing amount of acquired data requires the development of efficient denoising algorithms that will enable the detection of gravitational-wave events embedded in low signal-to-noise-ratio (SNR) environments. In this paper, an algorithm based on the local polynomial approximation (LPA) combined with the relative intersection of confidence intervals (RICI) rule for the filter support selection is proposed to denoise the gravitational-wave burst signals from core collapse supernovae. The LPA-RICI denoising method’s performance is tested on three different burst signals, numerically generated and injected into the real-life noise data collected by the Advanced LIGO detector. The analysis of the experimental results obtained by several case studies (conducted at different signal source distances corresponding to the different SNR values) indicates that the LPA-RICI method efficiently removes the noise and simultaneously preserves the morphology of the gravitational-wave burst signals. The technique offers reliable denoising performance even at the very low SNR values. Moreover, the analysis shows that the LPA-RICI method outperforms the approach combining LPA and the original intersection of confidence intervals (ICI) rule, total-variation (TV) based method, the method based on the neighboring thresholding in the short-time Fourier transform (STFT) domain, and three wavelet-based denoising techniques by increasing the improvement in the SNR by up to 118.94% and the peak SNR by up to 138.52%, as well as by reducing the root mean squared error by up to 64.59%, the mean absolute error by up to 55.60%, and the maximum absolute error by up to 84.79%.

## 1. Introduction

The first detection of a gravitational-wave signal from a compact binary coalescence (CBC) system [1,2] was made in 2015 by the Advanced LIGO (Laser Interferometer Gravitational-Wave Observatory) detectors [3]. This detection marked a turning point in gravitational-wave astronomy and initiated intensive scientific research in the field of gravitational-wave data analysis leading to the Nobel Prize in Physics in 2017. Besides the Advanced LIGO detectors, the Advanced Virgo detector [4,5] is operated by the European Gravitational Observatory (EGO) near Pisa, Italy. In addition to the first observation, the first two Advanced LIGO and Advanced Virgo observing runs (O1 and O2) resulted in observations of nine more binary black hole (BBH) mergers and one binary neutron star (BNS) merger [6]. The third run (O3a and O3b) was even more productive, with dozens of BNS, BBH, and neutron star-black hole (NSBH) observations. Moreover, the future increase in the detection rate is expected due to the detector sensitivity improvements and new detectors (LIGO-India and Kamioka Gravitational Wave Detector (KAGRA) [7] in Japan) joining the observation network [8]. The huge amount of acquired data requires the development of different specialized data processing methods for accurate identification of gravitational-wave events buried in instrumental and environmental noise. These will enable scientists to utilize observed gravitational-wave events in order to gain insights into the astrophysical origins and properties of different gravitational-wave sources and to test the consistency of the obtained data with those predicted by general relativity. Moreover, it will allow measurements of various cosmological parameters and may enable the detection of gravitational-waves from new types of sources, such as core collapse supernovae (CCSNs).

Gravitational-wave detectors are located at mutually distant sites (LIGO Hanford in Washington, USA; LIGO Livingston in Louisiana, USA; and Virgo in Pisa, Italy), and their main role is to detect gravitational-wave phenomena embedded in environmental and local instrumental noise and to calculate their polarization and source location. Each LIGO site runs an L-shaped Advanced LIGO detector [3] that consists of two orthogonal arms, where each arm is 4 km long ($L=L_{1}=L_{2}=4$ km) with two light-reflecting mirrors at each end. At the Advanced Virgo site [9], the detector’s arms are 3 km long. The gravitational-wave propagating through the gravitational-wave detector stretches one arm, while shortening the other. This difference in arm lengths, $\Delta L\left(t\right)=\delta L_{1}-\delta L_{2}$, causes the phase difference between two light fields returning to the beam splitter, which is recorded by the output photodetector, thus obtaining the optical signal proportional to the gravitational-wave strain amplitude h(t), defined as ΔL(t)=h(t)L [1].

The Advanced LIGO and Advanced Virgo detectors, in their most sensitive state, should be able to detect a change in distance 1/10,000th the width of a proton. In order to achieve their high sensitivity, detectors include several improvements of the Michelson interferometer, on which they are based. The first improvement refers to the size of the interferometers, which are the largest ever built. The longer the interferometer’s arms, the smaller the changes in the arm length that can be detected. Additionally, the basic Michelson interferometer is modified by including Fabry–Perot cavities. These optical cavities are formed by placing additional mirrors in the arms near the beam splitter, thus causing multiple laser reflections. These reflections increase the distance traveled by the laser beams by 300 times and build up the laser light, thus increasing the interferometer’s sensitivity [1,10,11].

Moreover, in order to increase the interferometer’s resolving power, the laser power must be increased from the input value of 40 W to the operating value of 750 kW. This is achieved by using power recycling mirrors at the input that continually reflect the laser light beams back into the Fabry–Perot cavities, thus boosting their power and sharpening the interference fringes [1,10,12]. Besides power recycling mirrors, the signal recycling mirrors are also used at the output to enhance the interference signal received by the photodetector. The interferometer operates a 1064 nm Nd:YAG laser in an ultrahigh vacuum system. The laser is stabilized in amplitude, frequency, and beam geometry to reduce the photon shot noise and maximize the conversion of gravitational strain to the optical signal [1,10,13,14].

In order to achieve high measurement sensitivity, the interferometer’s mirrors (test masses) must be isolated from seismic noise and designed in a way that reduces thermal noise. Different environmental sources produce vibrations and displacement noise that may affect test masses and sensitive measurements. In order to eliminate unwanted vibrations, the LIGO and Virgo sites employ different active and passive damping systems. The active damping system, called the internal seismic isolation (ISI) system, consists of position- and vibration- sensors that sense a range of frequencies characteristic of different environmental vibrations. The sensor data are processed in a control system that generates counter-movements of permanent-magnet actuators in order to cancel these vibrations. The passive damping is achieved by suspending each test mass by 0.4 mm fused-silica fibers at the end of a quadruple-pendulum system [1,15,16,17]. Thermal noise is reduced using materials with low mechanical losses for the test masses and suspensions [18,19,20].

Despite applying the above-described state-of-the-art noise reduction equipment, several potential noise sources could affect the background estimation of gravitational-wave events. Narrowband instrumental noises are caused by power lines (60 Hz and the associated harmonics), mechanical resonances of the system, and injected calibration signals [21]. Other potential instrumental noise sources include thermal noises, quantum noise, gas noise, charging noise, laser intensity and frequency fluctuation noise, RF oscillator noise, beam jitter, and electronics noise [21]. The uncorrelated environmental noise sources include noise sources associated with human activity that produces vibrational or acoustic noise, seismic waves from earthquakes (0.03–0.1 Hz), magnetic influences, malfunctions of the electro-optic modulator driver system (10–2000 Hz), and blip transients (30–250 Hz) [22,23]. Correlated noise sources are those that affect the detectors almost simultaneously, where electromagnetic sources include lightning strikes, solar events, and radio-frequency (RF) communication [22]. Thus, the gravitational-wave detector is characterized by non-white, non-stationary, and non-Gaussian noise. Moreover, gravitational-waves as astrophysical signals have typical amplitudes comparable to the detector background noise.

Therefore, the crucial research effort in gravitational-wave data analysis is the development of efficient denoising algorithms that will enable the detection of events embedded in low signal-to-noise-ratio (SNR) environments. Specific algorithms have been developed for different types of signals. Matched filtering was applied for the detection of CBC signals, i.e., signals from BBH mergers or signals from BNS [24,25,26]. This technique performed a search for CBC signals in the noisy detector data by correlating the data with a bank of generic transient signals or analytic waveform templates spanning a large astrophysical parameter space. However, matched filtering is optimal only for Gaussian noise, while detector noise is non-Gaussian and non-stationary. Moreover, modeling continuous gravitational-wave sources, such as spinning neutron stars, requires extensive computational resources, thus rendering the matched filtering method impractical for this type of signal. Continuous gravitational-wave signals were mainly identified using coherent detection methods and cross-correlating the data from multiple detectors [27,28].

Numerical-relativity simulations of gravitational-wave transients (bursts), such as CCSN signals, require significant computational efforts. Therefore, the models that could potentially be used in the above-mentioned identification methods are imperfect. Studies such as [29,30,31,32,33] proposed different approaches to estimating the physical parameters of this type of signal and their reconstruction from noisy data. These approaches were based on the combination of principal component analysis (PCA) and Bayesian data analysis techniques. The unmodeled long-lived burst signals were detected and reconstructed in [34,35] using coherent methods and a network of detectors. Burst signals of a short duration require specific pipelines that are able to provide differentiation between signal transients and detector noise glitches, such as the BayesWave [36], coherentWaveBurst [37], and oLIB [38] pipelines.

Machine learning-based algorithms have also gained attention recently with their applications in gravitational-wave detection and extraction from noisy data [39]. In [40], machine learning methods based on the dictionaries built from numerical-relativity templates of gravitational-wave signals were applied for data denoising, with satisfactory results for signals embedded in simulated Gaussian noise and some promising results for application on real gravitational-wave signals. Deep learning was applied in [41,42] for the noise reduction in the gravitational-wave detector data. The authors in [43] proposed deep filtering, which utilized deep learning with convolutional neural networks (CNNs) for detection and parameter estimation of gravitational-waves from BBH mergers, with signals being embedded in actual LIGO noise. The application of deep CNNs in detecting CBC signals was also assessed in several studies, with different neural network configurations applied to simulated signals corrupted by either synthetic or real noise [44,45,46,47]. The study in [48] presented the deep transfer learning method with deep CNNs for glitch classification and automatic clustering of new classes of anomalies occurring in data from the gravitational-wave detectors. Transient noise glitch classification necessary for interferometer characterization was also studied in [49] where deep CNNs were used to classify simulated glitches based on their time-frequency representations in the form of images. Detection and classification of noise transients were further studied in [50,51,52,53,54].

However, the aforementioned machine learning-based algorithms require extensive databases of different gravitational-wave signal templates. In the case of burst signals produced by core collapse, the generation of such databases that would be large enough is unfeasible due to the computational efforts needed to calculate such waveforms.

Therefore, the noise removal methods that do not require any a priori information about the underlying gravitational-wave signals, such as their signal morphology and astrophysical source, also have interesting potential applications. In [55], total-variation (TV)-based algorithms were applied for denoising of two types of gravitational-wave signals: signals from BBH mergers and burst signals produced by the CCSN. TV denoising methods, mainly applied in the field of image processing, are based on the $L_{1}$-norm minimization and Rudin–Osher–Fatemi (ROF) variational model [56,57,58,59,60,61,62,63]. The application of the TV denoising method in [55] resulted in successful noise removal. However, the study was limited to signals corrupted by the idealized additive Gaussian noise. This work was extended in [64] where the TV-based denoising method was successfully applied for denoising of gravitational-wave signals embedded in real noise data acquired from Advanced LIGO detectors, providing a detailed analysis of the model regularization parameter selection.

In this paper, we propose an algorithm based on the relative intersection of confidence intervals (RICI) rule combined with the local polynomial approximation (LPA) for denoising of gravitational-wave burst signals. The LPA is used as a filter design method in which a polynomial is fitted locally to the noisy measurement data within a data-driven, varying sliding window. The adaptive window size is selected by employing the asymmetrical RICI rule, which represents an improvement of the intersection of confidence intervals (ICI) rule. The LPA-RICI algorithm provides nearly optimal filter supports in terms of minimizing the estimation mean squared error (MSE). It does not require any information on the input signal, the noise, or the estimation of the bias, but only the noise variance estimation. This easy-to-implement algorithm is locally adaptive to the unknown and varying smoothness of the signal, with the estimation accuracy close to the one obtained when the original signal’s smoothness is known in advance.

We apply the LPA-RICI algorithm to the denoising of gravitational-wave bursts from CCSNs. The data are obtained by injecting numerically generated signals into the real-life non-Gaussian and non-stationary noise data obtained by the Advanced LIGO Livingston detector. The denoising procedure is performed for three different CCSN burst signals at three different distances (5, 10, and 20 kpc) corresponding to different (low) SNR levels. The numerical analysis done on the experimental results indicates that the proposed denoising method provides an accurate estimation of the original gravitational-wave signal corrupted by real-life noise data, by efficiently removing the noise and simultaneously preserving the characteristic features of CCSN bursts. Moreover, the LPA-RICI method outperforms several competing and conventionally applied denoising techniques, suggesting that it may be successfully applied in the preprocessing of gravitational-wave data characterized by intensive noise. The rest of the paper is organized as follows. Section 2 provides the theoretical background and mathematical framework of the LPA method used for the filter design and the RICI algorithm used for filter support selection. In Section 3, the experimental results obtained by several case studies are presented and discussed. Finally, the paper’s conclusion is given in Section 4.

## 2. Materials and Methods

Let us consider the noise-corrupted signal x(k), composed of the noise-free signal s(k) and the additive white Gaussian noise, $\eta \left(k\right)∼N\left(0,\sigma_{\eta }^{2}\right)$,

(1) x(k)=s(k)+η(k).

The goal of the signal denoising is to estimate $\hat{s}\left(k\right)$ from the noisy measurements x(k), such that the estimate $\hat{s}\left(k\right)$ is as close to s(k) as possible, i.e., the estimation error is minimized. This results in the instantaneous slope changes and other features in s(k) being well preserved. In order to achieve this goal, we applied the LPA method [65,66,67,68,69] as the filter design technique and proposed the adaptive RICI algorithm for the filter support selection.

### 2.1. The LPA Filter Design Method

The LPA method provides the estimate $\hat{s}\left(k\right)$ from the noisy measurements x(k), defined in (1), by fitting a polynomial to measurement data within a sliding window defined in the vicinity of the considered measurement. The polynomial, obtained as a linear combination of basis vectors, is fitted locally for each considered measurement so that it minimizes the following loss function using the weighted least squares (WLS) criterion [65,66,67,68]:

(2) $$\begin{array}{c} \begin{array}{cc} J_{LPA}\left(k_{0},w,C\right) & =\sum_{k=1}^{N_{k}}\psi_{w}\left(k-k_{0}\right){\left(x\left(k\right)-C^{T}\Phi_{w}\left(k-k_{0}\right)\right)}^{2}\\ \Phi (k) & ={\left[1,k,\frac{k^{2}}{2},\cdots ,\frac{k^{n-1}}{(n-1)!}\right]}^{T}\\ C & ={\left(C_{0},C_{1},C_{2},\cdots ,C_{n-1}\right)}^{T}, \end{array} \end{array}$$

where $k_{0}$ is the point of interest (center of the LPA), *k* is a signal sample, $N_{k}$ is the signal length, $\psi_{w}\left(k\right)$ is the scaled window function, *w* is the window size (filter support size), *C* is a vector of the polynomial coefficients, $\Phi_{w}\left(k\right)$ is a polynomial basis vector, and *n* is the order of the LPA.

The window function $\psi_{w}\left(k\right)$ defines the location of the polynomial fitting with respect to the central point $k_{0}$ and, in normalized form, satisfies the conventional kernel properties [65,66]:

(3) $$\psi \left(k\right)\geq 0;\;\psi \left(0\right)=\max_{k}\left\{\psi (k)\right\};\;\psi \left(k\right)\to 0\;as\;\left|k\right|\to \infty ;\;\int_{-\infty }^{+\infty }\psi \left(u\right)du=1$$

The minimization of criterion $J_{LPA}\left(k_{0},w,C\right)$, defined in (2), with respect to *C*, leads to the coefficient $\hat{C}\left(k_{0},w\right)$ [65,67]:

(4) $$\hat{C}\left(k_{0},w\right)=\underset{C\in R^{n}}{\mathrm{argmin}}J_{LPA}\left(k_{0},w,C\right)=\hat{s}\left(k_{0},w\right),$$

which represents the estimate of $s(k_{0})$ with respect to a window function of size *w*.

The estimates may be represented in the following form [67]:

(5) $$\hat{s}\left(k,w\right)=\sum_{k}q\left(k,k_{0},w\right)x\left(k\right),$$

where $q(k,k_{0},w)$ is the estimator kernel defined as [67]:

(6) $$q\left(k,k_{0},w\right)=\psi_{w}\left(k-k_{0}\right)\Phi_{w}^{T}\left(0\right)\Phi_{w}^{-1}\Phi_{w}\left(k-k_{0}\right),$$

with matrix $\Phi_{w}$ defined as:

(7) $$\Phi_{w}=\sum_{k}\psi_{w}\left(k-k_{0}\right)\Phi_{w}\left(k-k_{0}\right){\left[\Phi_{w}\left(k-k_{0}\right)\right]}^{T}.$$

The window function $\psi_{w}\left(k\right)$ is a scaled version of the normalized function defined in (3), where the scaling parameter w>0 represents the window length (filter support size) [65]:

(8) $$\psi_{w}\left(k\right)=\frac{1}{w}\psi \left(\frac{k}{w}\right).$$

The type of window function affects the weights associated with the signal samples, taken into consideration by local polynomial fitting inside the sliding window. Namely, the rectangular window function provides the same weights for all signal samples. In contrast, non-rectangular functions use higher weights for samples closer to the considered point $k_{0}$ and smaller weights for these farther away from the point of interest [68].

The filter support size *w* controls denoising quality and the smoothness of the signal estimate $\hat{s}\left(k,w\right)$: larger values of *w* signify including more samples in the LPA procedure, which leads to the increase of the estimation bias (and at the same time, the decrease in estimation variance), while smaller values of *w* cause the estimation variance to increase (as well as a smaller bias) [65,66]. Therefore, the selection of filter support size *w* is a crucial part of the denoising procedure. The main goal is to find such *w* that provides the optimal trade-off between estimation bias and variance, which is here done by applying the RICI algorithm.

### 2.2. The RICI Algorithm

The absolute value of the estimation error $\varepsilon_{m}\left(k,w\right)$ obtained by LPA estimators is defined as:

(9) $$\left|\varepsilon_{m}\left(k,w\right)\right|=\left|s\left(k\right)-{\hat{s}}_{m}\left(k,w\right)\right|,$$

where ${\hat{s}}_{m}\left(k,w\right)$ denotes the estimate of the signal sample value calculated using *m* samples in the vicinity of the considered sample and w(k) is the adaptive filter support size.

The point-wise mean squared estimation error, ρ(k,w), is given as [66,67]:

(10) $$\rho \left(k,w\right)=E\left\{\varepsilon_{m}^{2}\left(k,w\right)\right\}={\left|b_{m}\left(k,w\right)\right|}^{2}+\sigma_{m}^{2}\left(k,w\right),$$

where $b_{m}\left(k,w\right)$ is the estimation bias and $\sigma_{m}\left(k,w\right)$ is the standard deviation of the estimate ${\hat{s}}_{m}\left(k,w\right)$.

According to (10), the performance of the LPA estimators strongly depends on the adaptive filter support size w(k). Therefore, the main task of the denoising procedure consists of finding such a filter support size, $w^{o}\left(k\right)$, that provides the optimal bias-variance trade-off, hence minimizing ρ(k,w) [66,67]:

(11) $$w^{o}\left(k\right)=\underset{w(k)}{\mathrm{argmin}}\;\rho \left(k,w\right),$$

(12) $$\rho^{o}\left(k,w\right)=\min\left(\rho \left(k,w\right)\right)=\rho \left(k,w^{o}\right)=\left(1+\kappa^{2}\right)\sigma_{m}^{2}\left(k,w^{o}\right),$$

where the proportion parameter κ is defined as:

(13) $$\kappa =\frac{b_{m}\left(k,w^{o}\right)}{\sigma_{m}\left(k,w^{o}\right)}.$$

In the case of LPA estimators, the estimation error is given in the following form [65,67,70]:

(14) $$\left|\varepsilon_{m}\left(k,w\right)\right|\leq b_{m}\left(k,w\right)+\left|\zeta_{m}\left(k,w\right)\right|,$$

where $\zeta_{m}\left(k,w\right)∼N\left(0,\sigma_{m}^{2}\left(k,w\right)\right)$ is the random error.

The following inequality holds true with the probability p=1−β [65,67]:

(15) $$\left|\zeta_{m}\left(k,w\right)\right|\leq \chi_{1-\beta /2}\cdot \sigma_{m}\left(k,w\right),$$

where $\chi_{1-\beta /2}$ is the (1−β/2)th quantile of the standard Gaussian distribution, N(0,1).

Combining (14) and (15) suggests that the following inequality holds true with the same probability *p* [65,66]:

(16) $$\left|\varepsilon_{m}\left(k,w\right)\right|\leq b_{m}\left(k,w\right)+\chi_{1-\beta /2}\cdot \sigma_{m}\left(k,w\right).$$

As shown in [66,67], based on the estimation bias and standard deviation properties, for $w\left(k\right)<w^{o}\left(k\right)$, we have that:

(17) $$b_{m}\left(k,w\right)\leq \kappa \cdot \sigma_{m}\left(k,w\right).$$

Using (16) and (17), the following inequalities for the estimation error are obtained [66,67,70]:

(18) $$\left|\varepsilon_{m}\left(k,w\right)\right|\leq \left(\kappa +\chi_{1-\beta /2}\right)\cdot \sigma_{m}\left(k,w\right),$$

(19) $$\left|\varepsilon_{m}\left(k,w\right)\right|\leq \Gamma \cdot \sigma_{m}\left(k,w\right),$$

where parameter Γ is defined as the ICI threshold value:

(20) $$\Gamma =\kappa +\chi_{1-\beta /2}.$$

With the same probability *p*, (19) can be expressed as:

(21) $${\hat{s}}_{m}\left(k,w\right)-\Gamma \cdot \sigma_{m}\left(k,w\right)\leq s\left(k\right)\leq {\hat{s}}_{m}\left(k,w\right)+\Gamma \cdot \sigma_{m}\left(k,w\right),$$

which introduces the confidence intervals, $\Delta_{m}\left(k,w\right)$, that contain the noise-free signal values s(k) with the confidence *p*, defined as [65,67,70]:

(22) $$\Delta_{m}\left(k,w\right)=\left[{\hat{s}}_{m}\left(k,w\right)-\Gamma \cdot \sigma_{m}\left(k,w\right),{\hat{s}}_{m}\left(k,w\right)+\Gamma \cdot \sigma_{m}\left(k,w\right)\right],\;m=1,\cdots ,M,$$

(23) $$\Delta_{m}\left(k,w\right)=\left[\Delta_{l,m}\left(k,w\right),\Delta_{u,m}\left(k,w\right)\right],\;m=1,\cdots ,M,$$

where parameter Γ represents the critical value of the confidence interval $\Delta_{m}\left(k,w\right)$, while $\Delta_{l,m}\left(k,w\right)$ and $\Delta_{u,m}\left(k,w\right)$ are the lower and the upper confidence limits, respectively.

The estimated signal value ${\hat{s}}_{m}\left(k,w\right)$ is calculated using the LPA-based filters. For instance, in the case of zero-order LPA, the estimate is obtained by averaging sample values in the neighborhood of the considered sample, where the number of samples that are taken into calculation is determined by the RICI algorithm.

The first stage of the RICI algorithm is the ICI rule [71], which, for each signal sample *k*, calculates a sequence of *M* growing filter support values [65,67,70]:

(24) $$W=\left\{w_{1},w_{2},\cdots ,w_{M}\right\},\;w_{1}<w_{2}<\cdots <w_{M},$$

and the accompanying confidence intervals $\Delta_{m}\left(k,w\right)$, defined in (23).

The ICI rule provides the filter support candidates $w_{m}\left(k\right)$, such that $w_{m}\left(k\right)\leq w^{-}\left(k\right)$. The support $w^{-}\left(k\right)$ denotes the largest support for which the intersection of the confidence intervals is still non-empty [65,67,70]:

(25) $$w^{-}\left(k\right)=\underset{w_{m}\left(k\right)}{\mathrm{argmax}}\left\{\cap_{m=1}^{M}\Delta_{m}\left(k,w_{m}\right)\neq \emptyset \right\}.$$

This condition is met if the following inequality still holds true [65,67,70]:

(26) $${\underline{\Delta }}_{u,m}\left(k,w\right)\geq {\bar{\Delta }}_{l,m}\left(k,w\right),$$

where ${\underline{\Delta }}_{u,m}\left(k,w\right)$ is the smallest upper and ${\bar{\Delta }}_{l,m}\left(k,w\right)$ the largest lower confidence interval limit, defined as:

(27) $${\underline{\Delta }}_{u,m}\left(k,w\right)=\min_{j=1,\cdots ,m}\Delta_{u,j}\left(k,w_{j}\right),$$

(28) $${\bar{\Delta }}_{l,m}\left(k,w\right)=\max_{j=1,\cdots ,m}\Delta_{l,j}\left(k,w_{j}\right).$$

Several approaches on the filter support selection using the RICI rule are feasible, i.e., the above-explained procedure may be applied to only one side of the considered sample (left or right) or to both sides (symmetrical or asymmetrical filter supports). In this paper, we chose the asymmetrical filter support selection where the RICI algorithm is applied independently to the left-hand and right-hand side of the considered sample *k* [72].

Calculations are done for each signal sample *k*, resulting in two sets of confidence intervals $\Delta^{l}\left(k,w\right)$ and $\Delta^{r}\left(k,w\right)$, one for the left-hand side and one for the right-hand side of the considered sample [72]:

(29) $$\Delta^{l}\left(k,w\right)=\left\{\Delta_{1}^{l}\left(k,w\right),\Delta_{2}^{l}\left(k,w\right),\cdots ,\Delta_{k}^{l}\left(k,w\right)\right\},$$

(30) $$\Delta^{r}\left(k,w\right)=\left\{\Delta_{1}^{r}\left(k,w\right),\Delta_{2}^{r}\left(k,w\right),\cdots ,\Delta_{N_{k}-k}^{r}\left(k,w\right)\right\}.$$

According to confidence intervals definition, given in (22), their widths decrease as the number of samples *k* used for their calculation increases: $\Delta_{1}^{l}\left(k,w\right)>\Delta_{2}^{l}\left(k,w\right)>\cdots >\Delta_{k}^{l}\left(k,w\right)$ and $\Delta_{1}^{r}\left(k,w\right)>\Delta_{2}^{r}\left(k,w\right)>\cdots >\Delta_{N_{k}-k}^{r}\left(k,w\right)$.

In accordance with (22) and (23), the confidence limits of the confidence intervals determined by the asymmetrical RICI procedure are calculated as [72]:

(31) $$\Delta_{l,m}^{l}\left(k,w\right)={\hat{s}}_{m}^{l}\left(k,w\right)-\Gamma \cdot \sigma_{m}^{l}\left(k,w\right),$$

(32) $$\Delta_{u,m}^{l}\left(k,w\right)={\hat{s}}_{m}^{l}\left(k,w\right)+\Gamma \cdot \sigma_{m}^{l}\left(k,w\right),$$

(33) $$\Delta_{l,m}^{r}\left(k,w\right)={\hat{s}}_{m}^{r}\left(k,w\right)-\Gamma \cdot \sigma_{m}^{r}\left(k,w\right),$$

(34) $$\Delta_{u,m}^{r}\left(k,w\right)={\hat{s}}_{m}^{r}\left(k,w\right)+\Gamma \cdot \sigma_{m}^{r}\left(k,w\right),$$

where $\Delta_{l,m}^{l}\left(k,w\right)$ ($\Delta_{u,m}^{l}\left(k,w\right)$) and $\Delta_{l,m}^{r}\left(k,w\right)$ ($\Delta_{u,m}^{r}\left(k,w\right)$) are the lower (upper) confidence limits of the confidence interval calculated using *m* adjacent noisy signal sample values (including the considered signal sample) to the left-hand side and to the right-hand side of the considered sample *k*, respectively; ${\hat{s}}_{m}^{l}\left(k,w\right)$ and ${\hat{s}}_{m}^{r}\left(k,w\right)$ are the estimates of the considered signal sample value, calculated using sample values to its left- and right-hand side, respectively; $\sigma_{m}^{l}\left(k,w\right)$ and $\sigma_{m}^{r}\left(k,w\right)$ are the standard deviations of the estimation error for the left- and right-hand side calculations, respectively.

The algorithm operates on the each side of the considered *k*th sample independently by tracking the intersection of the currently calculated *m*th confidence interval ($\Delta_{m}^{l}\left(k,w\right)=\left[\Delta_{l,m}^{l}\left(k,w\right),\Delta_{u,m}^{l}\left(k,w\right)\right]$ for the left- and $\Delta_{m}^{r}\left(k,w\right)=\left[\Delta_{l,m}^{r}\left(k,w\right),\Delta_{u,m}^{r}\left(k,w\right)\right]$ for the right-hand side) with the intersection of all previous m−1 confidence intervals (denoted as ${\bar{\underline{\Delta }}}_{m-1}^{l}\left(k,w\right)$ for the left- and ${\bar{\underline{\Delta }}}_{m-1}^{r}\left(k,w\right)$ for the right-hand side). If the confidence intervals’ intersection is non-empty, the algorithm marks $w_{m}^{l}\left(k\right)$ and $w_{m}^{r}\left(k\right)$ as the current candidate for the optimal filter support to the left- and to the right-hand side of the considered sample, respectively. This intersection condition is checked by comparing the values of the smallest upper ${\underline{\Delta }}_{u,m}^{l}\left(k,w\right)$ and the largest lower ${\bar{\Delta }}_{l,m}^{l}\left(k,w\right)$ confidence limit on the left side [72]:

(35) $${\underline{\Delta }}_{u,m}^{l}\left(k,w\right)\geq {\bar{\Delta }}_{l,m}^{l}\left(k,w\right),$$

and the values of the smallest upper ${\underline{\Delta }}_{u,m}^{r}\left(k,w\right)$ and the largest lower ${\bar{\Delta }}_{l,m}^{r}\left(k,w\right)$ confidence limit on the right side:

(36) $${\underline{\Delta }}_{u,m}^{r}\left(k,w\right)\geq {\bar{\Delta }}_{l,m}^{r}\left(k,w\right).$$

The above described first stage of the RICI algorithm (the ICI stage) results in $w^{l-}\left(k\right)$ for the left-hand side calculations and $w^{r-}\left(k\right)$ for the right-hand side calculations, as the largest filter supports satisfying (35) and (36), respectively. Finally, the largest candidate for the optimal filter support $w^{-}\left(k\right)$ is obtained as [72]:

(37) $$w^{-}\left(k\right)=w^{l-}\left(k\right)+w^{r-}\left(k\right)-1,$$

where the fact that both supports (left and right) contain the considered sample *k* is taken into account.

The thus obtained filter support $w^{-}\left(k\right)$ is close to the optimal value $w^{o}\left(k\right)$, defined in (11) [70,73].

The performance of the described ICI stage relies heavily on the proper selection of the threshold parameter Γ. Namely, selecting too small Γ values leads to $w^{-}\left(k\right)<w^{o}\left(k\right)$, thus resulting in signal undersmoothing, while selecting Γ too large results in signal oversmoothing, as $w^{-}\left(k\right)>w^{o}\left(k\right)$ [65,66]. Moreover, the ICI stage shows weaker performance when applied to wide regions of nearly constant signal values in which sudden changes occur [72].

Therefore, the second stage of the algorithm, the RICI rule stage, introduces the additional criterion for the adaptive filter support selection and applies it to the filter support candidates obtained in the first stage (the ICI rule stage) of the algorithm. The RICI rule method significantly improves the estimation accuracy of the ICI rule method for the same values of parameter Γ, while simultaneously being more robust to suboptimal Γ values [72,74,75]. The study in [74] considered only high SNR scenarios of the synthetic data corrupted by the additive white Gaussian noise. In this paper, the approach is extended to the real-life problem of gravitational-wave denoising using different orders of the LPA in the intensive noise scenarios (low SNRs), characterized by the non-stationary and non-Gaussian noise.

The RICI criterion is defined with respect to the ratio of the intersection of the confidence intervals’ width and the current confidence interval’s width, thus taking into account the relative amount of confidence intervals overlapping [72,74]. As in the previous algorithm stage, this ratio is also calculated for both sides of the considered sample, thus obtaining $R_{m}^{l}\left(k,w\right)$ for the left-hand side [72]:

(38) $$R_{m}^{l}\left(k,w\right)=\frac{{\underline{\Delta }}_{u,m}^{l}\left(k,w\right)-{\bar{\Delta }}_{l,m}^{l}\left(k,w\right)}{\Delta_{u,m}^{l}\left(k,w\right)-\Delta_{l,m}^{l}\left(k,w\right)}=\frac{{\underline{\Delta }}_{u,m}^{l}\left(k,w\right)-{\bar{\Delta }}_{l,m}^{l}\left(k,w\right)}{2\Gamma \sigma_{m}^{l}\left(k,w\right)},$$

and $R_{m}^{r}\left(k,w\right)$ for the right-hand side:

(39) $$R_{m}^{r}\left(k,w\right)=\frac{{\underline{\Delta }}_{u,m}^{r}\left(k,w\right)-{\bar{\Delta }}_{l,m}^{r}\left(k,w\right)}{\Delta_{u,m}^{r}\left(k,w\right)-\Delta_{l,m}^{r}\left(k,w\right)}=\frac{{\underline{\Delta }}_{u,m}^{r}\left(k,w\right)-{\bar{\Delta }}_{l,m}^{r}\left(k,w\right)}{2\Gamma \sigma_{m}^{r}\left(k,w\right)}.$$

The RICI criterion is defined for the left-hand side of the considered sample as [72]:

(40) $$R_{m}^{l}\left(k,w\right)\geq R_{c},$$

and for the right-hand side as:

(41) $$R_{m}^{r}\left(k,w\right)\geq R_{c},$$

where $R_{c}$ is a preset threshold value ($0\leq R_{c}\leq 1$).

The RICI stage results in $w^{l+}\left(k\right)$ as the largest filter support satisfying (35) and (40) for the left-hand side calculations, while $w^{r+}\left(k\right)$ is obtained by the right-hand side calculations, as the largest filter support satisfying (36) and (41) [72].

Finally, the filter support $w^{+}\left(k\right)$ obtained by the RICI algorithm is calculated as:

(42) $$w^{+}\left(k\right)=w^{l+}\left(k\right)+w^{r+}\left(k\right)-1.$$

## 3. Results and Discussion

### 3.1. Data Conditioning

In order to test whether the proposed LPA-RICI-based denoising algorithm can efficiently suppress noise in gravitational-wave data in real-life conditions, numerically generated CCSN burst signal templates are injected into the real-life noise data collected by the Advanced LIGO Livingston detector. Burst signals are obtained from the Dimmelmeier catalog [76], which contains waveforms generated by general-relativistic simulations of rotating stellar core collapse neutron stars. We assess the denoising performance of the LPA-RICI method on three CCSN template signals: s20a1o05, s20a2o09, and s20a3o15 (data publicly available at https://zenodo.org/record/4108838#.X48R7pxR1Pa), as in [64]. Moreover, the denoising performance for each signal is analyzed when the signal source is located at three different distances (5, 10, and 20 kpc), which correspond to different SNR levels.

As the Advanced LIGO detector noise is non-Gaussian and non-stationary, the data are first preprocessed using the autoregressive model developed in [77,78], in order to whiten the data, i.e., to transform the colored noise into the white noise that is flat in frequency. Afterward, the LPA-RICI denoising algorithm is applied.

#### Whitening Procedure

The data whitening procedure is applied to make the data delta-correlated. This means that the sequence of data x[n], after the whitening procedure, will be uncorrelated at each lag different from zero, i.e., the autocorrelation $r_{xx}\left[n\right]$ is a delta function. There are several strategies to perform this process: some are based on techniques in the frequency domain, others on techniques in the time domain. The result of this operation is that the contribution of the statistics up to the second order will be removed from the data. In this work, we used the time domain technique, developed in [77,79], which is based on the time domain procedure, using an autoregressive (AR) fit to the data. An autoregressive process x[n] of order *P* with parameter $a_{k}$, hereafter AR(P), is characterized by the relation:

(43) $$x\left[n\right]=\sum_{k=1}^{P}a_{k}x\left[n-k\right]+\sigma w\left[n\right],$$

where w[n] is a white Gaussian process.

The problem of determining the AR parameters is the same as that of finding the optimal “weights vector” $w=w_{k}$, for k=1,...P, for the linear prediction problem [80]. In the linear prediction, we would predict the sample x[n] using the *P* previous observed data x[n]={x[n−1],x[n−2],⋯,x[n−P]}, building the estimate as a transversal filter:

(44) $$\hat{x}\left[n\right]=\sum_{k=1}^{P}w_{k}x\left[n-k\right].$$

We can find the coefficients of the linear predictor by minimizing a cost function that is the mean squared error $\epsilon =E[e{\left[n\right]}^{2}]$ (E is the ensemble average operator), with:

(45) $$e\left[n\right]=x\left[n\right]-\hat{x}\left[n\right]$$

Being the error we make in this prediction and obtaining the so-called normal or Wiener–Hopf equations:

(46) $$\epsilon_{min}=r_{xx}\left[0\right]-\sum_{k=1}^{P}w_{k}r_{xx}\left[-k\right],$$

which are identical to the Yule–Walker equations [80] used to estimate the AR parameters $a_{k}$ from the autocorrelation function, with $w_{k}\;=\;-a_{k}$ and $\epsilon_{min}=\sigma^{2}$.

This is the key relationship between the AR model and the linear prediction, which assures obtaining a filter that is stable and causal [80]. In this way, we can use the AR model to reproduce stable processes in the time domain.

What we have to do is to simply find the *P* parameters that fit the power spectral density of our process, and at the same time, we find the optimal weights vector that allows us to reproduce the process at time *n* knowing the process at the previous time *P*. The method that uses this estimation tries to make the error signal (45) a white process. This is the reason we call this a whitening procedure.

Using a lattice structure [78], we can implement the whitening filter in the time domain. This is a procedure that is used for other pipelines [50,81] and that can eventually be also implemented in an adaptive way [77], taking care of the non-stationary noise.

### 3.2. Data Denoising

The results obtained by the LPA-RICI denoising of gravitational-wave data are also compared to the results obtained by several conventionally applied signal denoising methods, including the LPA-ICI method [65], the TV-L1 denoising method with the primal-dual algorithm [56,82,83], the method based on the neighboring thresholding in the short-time Fourier transform (STFT) domain (Neigh STFT) [84], and three wavelet denoising methods. The data-driven Neigh STFT method represents an adaptive noise level estimation and denoising algorithm based on the minimal controlled recursive averaging estimator and neighboring block thresholding in the STFT domain, where the optimal threshold and block size are automatically adjusted by minimizing Stein’s unbiased risk estimator (SURE) [84]. An analysis of the wavelet-based denoising techniques was conducted by inspecting the denoising performances of three different wavelets—symlet wavelets, Daubechies wavelets, and coiflet wavelets. For each wavelet, a range of the numbers of vanishing moments was considered (sym2-sym45, db1-db45, and coif1-coif5), as well as different levels of wavelet decomposition and threshold selection rules applied to the wavelet coefficients (including SURE [85] and minimax [86] thresholding). These wavelet-based methods employ hard thresholding and multiplicative threshold rescaling using a level-dependent estimation of level noise.

For each tested denoising method, the optimal algorithm parameters are selected by conducting an extensive search in the parameter space and choosing parameter values that minimize the estimated root mean squared error (RMSE). In order to quantify the performance of the proposed LPA-RICI-based denoising algorithm in comparison to the alternative methods, a set of performance indices is used.

### 3.3. Performance Indices

The estimation efficiency of the LPA-RICI-based gravitational-wave denoising is assessed using the following performance indices:

- Improvement in the signal-to-noise ratio (ISNR):
  (47) $$\mathrm{ISNR}=10\log_{10}\left(\frac{\sum_{k=1}^{N_{k}}{\left(s(k)-x(k)\right)}^{2}}{\sum_{k=1}^{N_{k}}{\left(s\left(k\right)-{\hat{s}}_{m}\left(k\right)\right)}^{2}}\right)$$
- Peak signal-to-noise ratio (PSNR):
  (48) $$\mathrm{PSNR}=20\log_{10}\left(\frac{\max_{k=1,\cdots ,N_{k}}s\left(k\right)}{\sqrt{\frac{1}{N_{k}}\sum_{k=1}^{N_{k}}{\left(s\left(k\right)-{\hat{s}}_{m}\left(k\right)\right)}^{2}}}\right)$$
- Root mean squared error (RMSE):
  (49) $$\mathrm{RMSE}=\sqrt{\frac{1}{N_{k}}\sum_{k=1}^{N_{k}}{\left(s\left(k\right)-{\hat{s}}_{m}\left(k\right)\right)}^{2}}$$
- Mean absolute error (MAE):
  (50) $$\mathrm{MAE}=\frac{1}{N_{k}}\sum_{k=1}^{N_{k}}\left|s\left(k\right)-{\hat{s}}_{m}\left(k\right)\right|$$
- Maximum absolute error (MAX):
  (51) $$\mathrm{MAX}=\max_{k=1,\cdots ,N_{k}}\left|s\left(k\right)-{\hat{s}}_{m}\left(k\right)\right|$$

Performance indices RMSE, MAE, and MAX are given as normalized values in the following subsection, i.e., they are calculated based on the normalized signals obtained by dividing the signals by their maximum values. This normalized representation facilitates the comparison of the performance indices’ values between different signals and corresponding SNR values.

### 3.4. Case Studies

#### 3.4.1. Case Study—Signal s20a1o05

**Figure 1:**
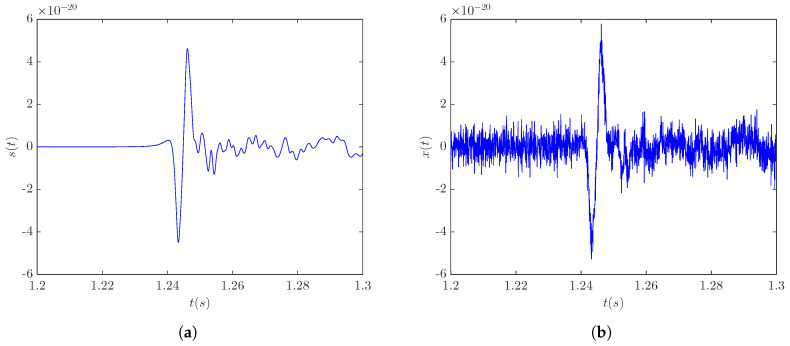
CCSN signal s20a1o05 at a distance of 5 kpc: (**a**) template signal; (**b**) noisy signal (SNR = 3.9 dB).

The denoising accuracy of the LPA-RICI method with the signal s20a1o05 was analyzed for three different signal source distances, namely, 5, 10, and 20 kpc, corresponding to the SNR levels of 3.9 dB, −2.11 dB, and −8.13 dB, respectively. CCSN burst template signal s20a1o05 at a distance of 5 kpc is shown in Figure 1a, while Figure 1b shows the noise-corrupted version of this signal. As seen in Figure 1, signal s20a1o05 and other signals of this type, including signal s20a2o09 and signal s20a3o15, are characterized by a negative peak whose occurrence is associated with the core bounce and is followed by damped oscillations of the proto-neutron star.

**Figure 2:**
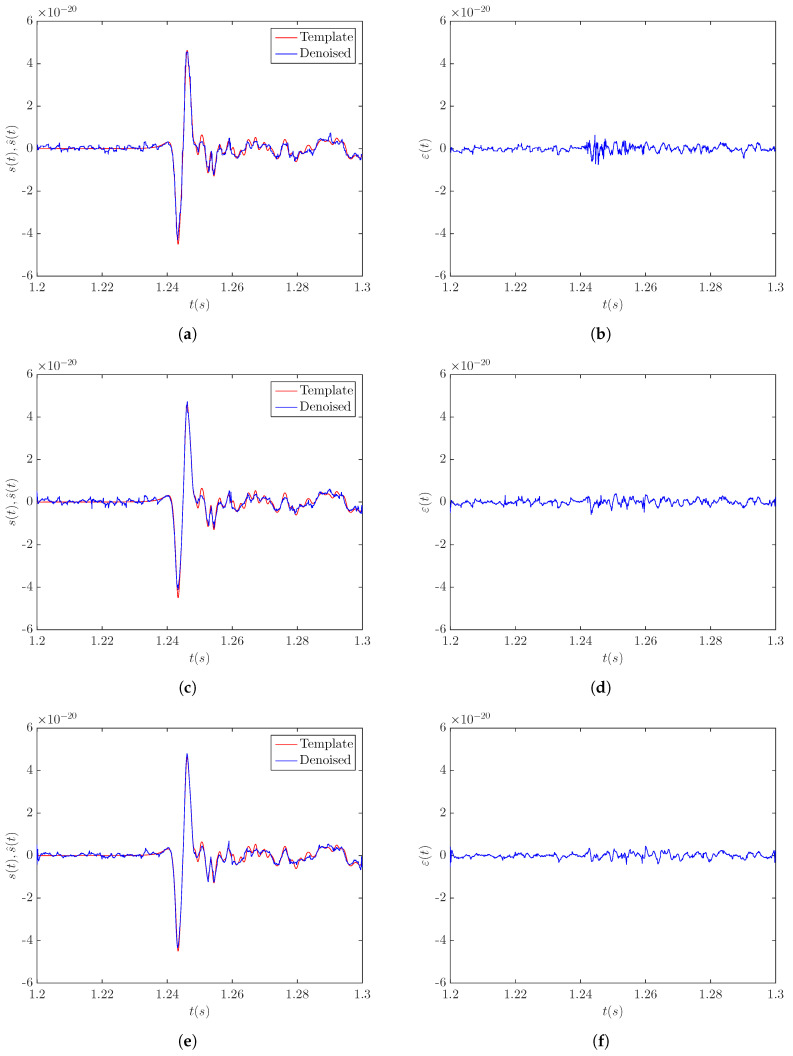
Results of applying the LPA-RICI denoising method to the noisy CCSN signal s20a1o05 at a distance of 5 kpc (SNR = 3.9 dB): (**a**) template and LPA-RICI denoised signal (*n* = 0, Γ = 5.5, *R_c_* = 1); (**b**) LPA-RICI estimation error (*n* = 0, Γ = 5.5, *R_c_* = 1); (**c**) template and LPA-RICI denoised signal (*n* = 1, Γ = 7, *R_c_* = 1); (**d**) LPA-RICI estimation error (*n* = 1, Γ = 7, *R_c_* = 1); (**e**) template and LPA-RICI denoised signal (*n* = 2, Γ = 11, *R_c_* = 1); (**f**) LPA-RICI estimation error (*n* = 2, Γ = 11, *R_c_* = 1).

The results obtained by applying the LPA-RICI denoising algorithm to the noisy signal s20a1o05 at a distance of 5 kpc are presented in Figure 2. Figure 2a,c,e shows the comparison between the original template signal and the signal obtained by the LPA-RICI denoising procedure with the LPA order set to the values of n=0, n=1, and n=2, respectively. Figure 2b,d,f shows the respective estimation errors. The results presented in Figure 2 suggest that the LPA-RICI method successfully removes the noise from the noisy signal s20a1o05 at a distance of 5 kpc, as the denoised signals fit the templates almost perfectly. All three LPA-RICI variants provide excellent denoising performance, with the second-order LPA variant producing the best results visually.

The quantitative comparisons of the denoising results obtained by the LPA-RICI method, the LPA-ICI technique, the TV method, the Neigh STFT technique, the symlet, the Daubechies, and the coiflet wavelet-based methods, for the signal s20a1o05 at distances of 5, 10, and 20 kpc, are given in Table 1, Table 2 and Table 3, respectively. Performance indices ISNR and PSNR are shown in dB, while RMSE, MAE, and MAX are given as normalized values. The best performance indices in each table, i.e., the highest values of ISNR and PSNR and the lowest values of RMSE, MAE, and MAX, are marked in bold. The results presented in Table 1, Table 2 and Table 3 confirm that all three variants of the proposed LPA-RICI method provide excellent denoising performance, improving the SNR of the signal and reducing the estimation errors. As the signal source distance increases (and the SNR of the noisy signal decreases), the performance of the LPA-RICI method deteriorates slightly, but still provides satisfactory denoising results. At all three distances, the best results are obtained when the second-order LPA is applied.

The relative performance improvement of the second-order LPA-RICI method over the other tested methods for the signal s20a1o05 at distances of 5, 10, and 20 kpc is calculated, and the percentage values are given in Table 4, Table 5 and Table 6, respectively. The positive percentage values indicate the performance improvement of the LPA-RICI method over the other denoising methods, i.e., the increase in the values of performance indices ISNR and PSNR and the decrease in the values of RMSE, MAE, and MAX. On the other hand, negative values indicate performance deterioration. The results presented in these tables suggest that the LPA-RICI method outperforms the other tested methods at each considered distance, by increasing ISNR by up to 118.94% and PSNR by up to 27.49%, as well as by reducing RMSE by up to 54.51%, MAE by up to 48.28%, and MAX by up to 72.85%. At 5 and 20 kpc, lower MAE values are provided by the db13 wavelet and the Neigh STFT method, respectively, but only by a small margin.

The execution times were also calculated for each tested denoising method. The algorithm execution times were obtained on a computer with the Intel Core i7-4720HQ CPU @ 2.60 GHz, and 8 GB of RAM. The results were averaged over 1000 algorithm runs. The algorithm execution times of each method applied to the denoising of the signal s20a1o05, at distances of 5, 10, and 20 kpc, are given in Table 7. The presented results suggest that the proposed LPA-RICI method outperforms, in terms of algorithm execution times, the original LPA-ICI method in all considered cases, and the Neigh STFT method in most cases. However, it exhibits weaker performance when compared to the TV-L1 method and wavelet-based techniques.

#### 3.4.2. Case Study—Signal s20a2o09

**Figure 3:**
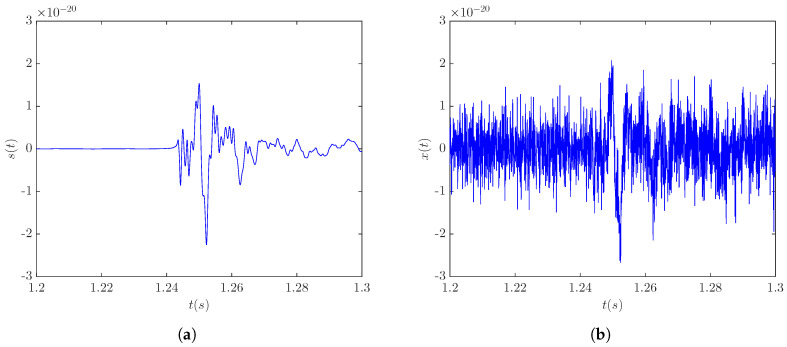
CCSN signal s20a2o09 at a distance of 5 kpc: (**a**) template signal; (**b**) noisy signal (SNR = −4.54 dB).

The denoising of the CCSN burst signal s20a2o09 was assessed for three different cases obtained by placing the signal source at three different distances. Distances of 5, 10, and 20 kpc correspond to the SNR levels of −4.54 dB, −10.09 dB, and −15.98 dB, respectively. Figure 3a shows the template signal s20a2o09 at a distance of 5 kpc, while Figure 3b shows the same signal embedded in the detector noise.

**Figure 4:**
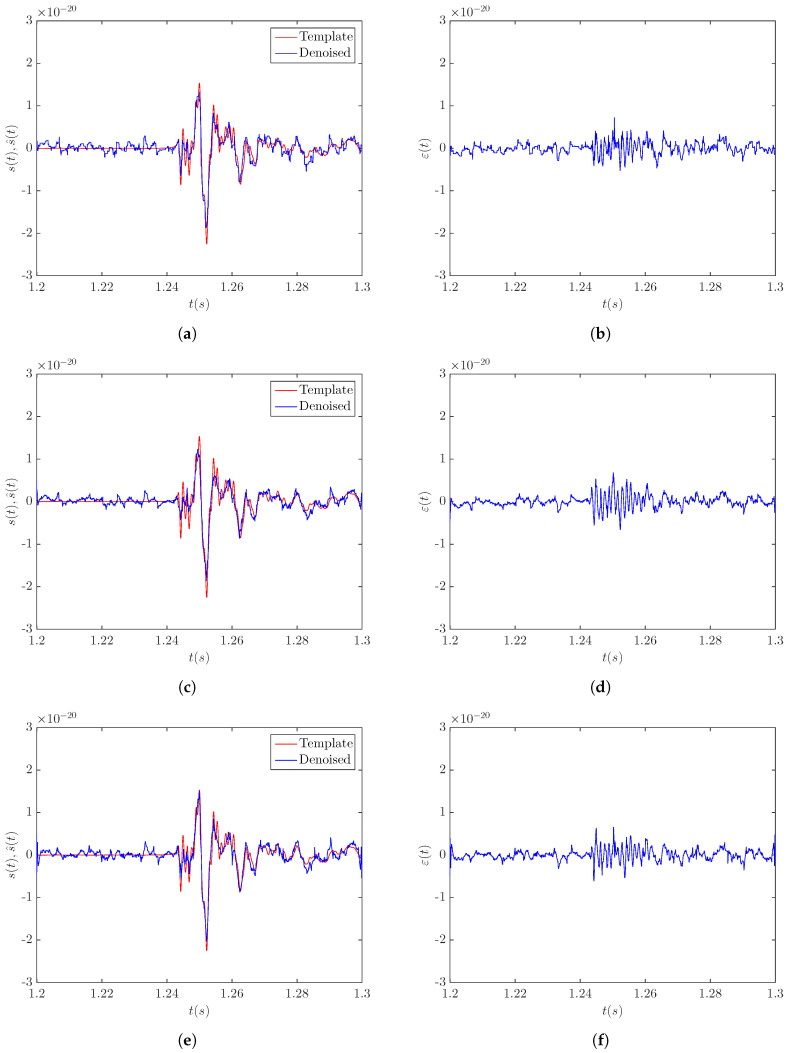
Results of applying the LPA-RICI denoising method to the noisy CCSN signal s20a2o09 at a distance of 5 kpc (SNR = −4.54 dB): (**a**) template and LPA-RICI denoised signal (*n* = 0, Γ = 9, *R_c_* = 1); (**b**) LPA-RICI estimation error (*n* = 0, Γ = 9, *R_c_* = 1); (**c**) template and LPA-RICI denoised signal (*n* = 1, Γ = 13, *R_c_* = 1); (**d**) LPA-RICI estimation error (*n* = 1, Γ = 13, *R_c_* = 1); (**e**) template and LPA-RICI denoised signal (*n* = 2, Γ = 16, *R_c_* = 1); (**f**) LPA-RICI estimation error (*n* = 2, Γ = 16, *R_c_* = 1).

Figure 4 shows the results obtained by the LPA-RICI denoising of the noisy signal s20a2o09 at a distance of 5 kpc. Figure 4a,c,e displays the comparison between the template signal and the signal obtained by the LPA-RICI denoising procedure with the LPA order set to the values of n=0, n=1, and n=2, respectively. The corresponding estimation errors are given in Figure 4b,d,f. As seen in Figure 4, the denoised signals obtained by all three LPA-RICI variants are very close to the templates. The main positive and negative peaks are well reconstructed, as well as subsequent oscillations. Therefore, the presented results suggest that the LPA-RICI method efficiently removes the noise from the noisy signal s20a2o09 at a distance of 5 kpc, while simultaneously preserving the signal morphology.

Table 8, Table 9 and Table 10 provide the values of denoising performance indices obtained by the LPA-RICI method and other tested methods applied to the noisy signal s20a2o09 at distances of 5, 10, and 20 kpc, respectively. The presented results indicate that all three variants of the applied LPA-RICI method provide good denoising performance, based on improving the noisy signal SNR and reducing the estimation errors. The LPA-RICI method’s denoising performance somewhat deteriorates as the distance is increased due to the very low SNR, but the obtained estimation results are still satisfactory.

Table 11 gives the relative change of the performance indices obtained by the second-order LPA-RICI method compared to those obtained by other denoising methods applied to the signal s20a2o09 at a distance of 5 kpc. As seen in Table 11, the LPA-RICI method outperforms the other tested denoising methods, except the TV method and the coif1 wavelet method. However, the performances of these three methods are very close. The results presented in Table 12 suggest that the second-order LPA-RICI method, applied to the denoising of the signal s20a2o09 at a distance of 10 kpc, outperforms the other tested methods, with the exception of the performance index MAE, which is slightly worse than those obtained by the LPA-ICI method and the TV method. Table 13 shows the comparison of performance indices obtained by the zero-order LPA-RICI method and performance indices obtained by other methods applied to the denoising of the signal s20a2o09 at a distance of 20 kpc. In this case, the LPA-RICI method outperforms the other methods, except the TV method, which is slightly better for performance indices ISNR, PSNR, and RMSE, but only by a very small margin.

To sum up, the LPA-RICI method applied to the denoising of the signal s20a2o09 at all three distances provides a denoising performance close to the one obtained by the TV method. It outperforms the other methods by increasing ISNR by up to 95.56% and PSNR by up to 132.60%, as well as by reducing RMSE by up to 62.13%, MAE by up to 55.60%, and MAX by up to 83.31%.

Table 14 presents the algorithm execution times of each method applied to the denoising of the signal s20a2o09, at distances of 5, 10, and 20 kpc. These results indicate that the LPA-RICI method is outperformed by the TV-L1 method and wavelet-based techniques in terms of algorithm execution times. However, it runs competitively with the Neigh STFT denoising method and significantly reduces the execution time when compared to the original LPA-ICI approach.

#### 3.4.3. Case Study—Signal s20a3o15

**Figure 5:**
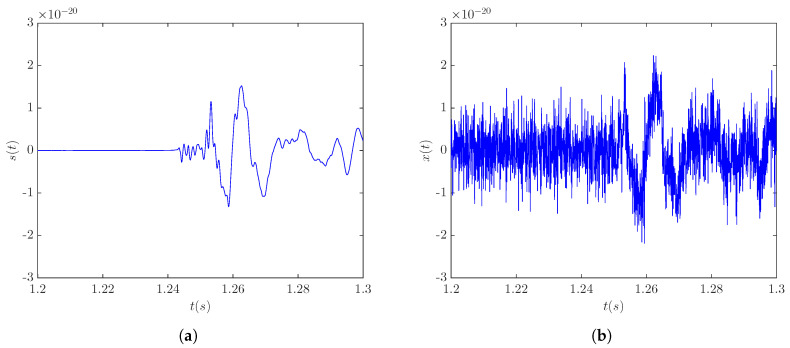
CCSNsignal s20a3o15 at a distance of 5 kpc: (**a**) template signal; (**b**) noisy signal (SNR = −2.18 dB).

The denoising procedure for the signal s20a3o15 was conducted at three different signal source distances: 5, 10, and 20 kpc, corresponding to the SNR levels of −2.18 dB, −8.17 dB, and −14.19 dB, respectively. The considered template signal s20a3o15 at a distance of 5 kpc is shown in Figure 5a, while Figure 5b shows the template signal corrupted by the LIGO detector noise.

**Figure 6:**
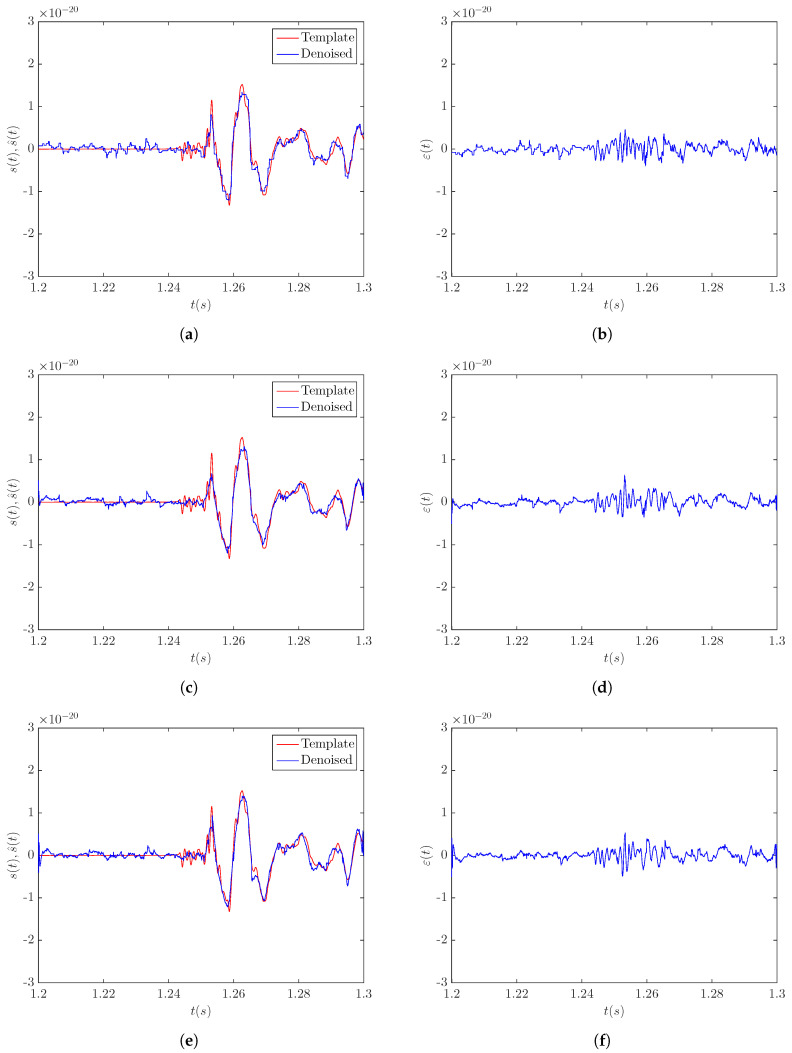
Results of applying the LPA-RICI denoising method to the noisy CCSN signal s20a3o15 at a distance of 5 kpc (SNR = −2.18 dB): (**a**) template and LPA-RICI denoised signal (*n* = 0, Γ = 10.75, *R_c_* = 1); (**b**) LPA-RICI estimation error (*n* = 0, Γ = 10.75, *R_c_* = 1); (**c**) template and LPA-RICI denoised signal (*n* = 1, Γ = 16, *R_c_* = 1); (**d**) LPA-RICI estimation error (*n* = 1, Γ = 16, *R_c_* = 1); (**e**) template and LPA-RICI denoised signal (*n* = 2, Γ = 20, *R_c_* = 1); (**f**) LPA-RICI estimation error (*n* = 2, Γ = 20, *R_c_* = 1).

The results obtained by applying the LPA-RICI method to the noisy signal s20a3o15 at a distance of 5 kpc are displayed in Figure 6. Figure 6a,c,e provides the comparison between the template signal and the denoised signal obtained by the LPA-RICI procedure with the LPA order set to the values of n=0, n=1, and n=2, respectively. Figure 6b,d,f shows the corresponding estimation errors. The denoised signals obtained by all three LPA-RICI variants fit the templates very well, indicating successful removal of the noise from the noisy signal s20a3o15 at a 5 kpc distance.

The comparisons of the results obtained by the LPA-RICI method and other tested methods for the denoising of the signal s20a3o15 at distances of 5, 10, and 20 kpc are given in Table 15, Table 16 and Table 17, respectively. The results presented in these three tables suggest that all three variants of the LPA-RICI method provide excellent denoising performance, significantly improving the SNR of the noisy signal and reducing the estimation errors. The denoising performance of the LPA-RICI method deteriorates slightly with the increasing distance due to the increased noise intensity, but this decline in estimation accuracy is less pronounced than with other methods, and the LPA-RICI still provides good denoising results even for a very low SNR value.

The percentage values of the relative estimation improvement of the LPA-RICI method over the other tested techniques for the signal s20a3o15 at distances of 5, 10, and 20 kpc are given in Table 18 (the first-order LPA), Table 19 (the zero-order LPA), and Table 20 (the zero-order LPA), respectively. The results presented in these tables suggest that the LPA-RICI method significantly outperforms the other tested methods at each considered distance and SNR level, by increasing ISNR by up to 100.07% and PSNR by up to 138.52%, as well as by reducing RMSE by up to 64.59%, MAE by up to 44.13%, and MAX by up to 84.79%.

The algorithm execution times of each technique applied to the denoising of the signal s20a3o15, at distances of 5, 10, and 20 kpc, are provided in Table 21. The presented results show that the wavelet-based techniques provide the best performance in terms of algorithm execution times, followed by the TV-L1 method. However, the proposed LPA-RICI method provides execution time performance that is in most cases competitive with the one obtained by the Neigh STFT denoising technique and significantly reduces the algorithm execution time when compared to the LPA-ICI approach.

To sum up the above discussion, we may point out that the proposed LPA-RICI method outperforms other tested competitive methods (the approach combining LPA and the original ICI rule, the TV-L1 method, the method based on the neighboring thresholding in the STFT domain, and the three wavelet-based denoising techniques) in gravitational-wave denoising in low SNR scenarios.

## 4. Conclusions

In this paper, the LPA-RICI algorithm is proposed to denoise the gravitational-wave burst signals from the CCSN. This data-driven, locally adaptive, and easy-to-implement method is applied to denoising numerically generated burst signals injected into the real-life non-Gaussian and non-stationary noise data obtained by the Advanced LIGO detector. The estimation accuracy of the LPA-RICI denoising method is assessed for three different burst signals, with each being placed at different distances corresponding to the different values of SNR. The analysis of the experimental results obtained by these case studies indicates that the LPA-RICI method efficiently suppresses the noise and simultaneously preserves the critical features (morphology) of the gravitational-wave burst signals. The approach provides good denoising performance even for signals corrupted by the intensive noise (very low SNR values). Moreover, the comparative analysis shows that the LPA-RICI method outperforms several conventionally applied denoising techniques of similar complexity (the original LPA-ICI approach, the TV-L1 method, the method based on the neighboring thresholding in the STFT domain, and the three wavelet-based denoising techniques). Therefore, the results obtained in this work suggest that the LPA-RICI method may be successfully applied in the preprocessing of the noisy gravitational-wave data collected by the Advanced LIGO and Advanced Virgo detectors and included as a part of the algorithm pipeline for the advanced detection and identification of gravitational-wave events.

The main challenges in this research field include detecting useful information and burst signals in intensive noise environments (low SNRs). This problem may also be approached as a classification problem, which can be solved by machine learning techniques. We plan to research this in the near future; namely, instead of analyzing noisy time-series, we want to implement Cohen’s class of time-frequency distributions and apply machine learning techniques to distinguish between signal and noise in the quadratic time-frequency representations. According to the literature review, this approach has not been used in the gravitational-waves field up to now.

## Figures and Tables

**Table 1:** Denoising results for the CCSN signal s20a1o05 at a distance of 5 kpc (SNR = 3.9 dB). The best performance indices are marked in bold.

Perform. Index	LPA-RICI n=0 Γ=5.5, $R_{c}=1$	LPA-RICI n=1 Γ=7, $R_{c}=1$	LPA-RICI n=2 Γ=11, $R_{c}=1$	LPA-ICI Γ=0.75	TV μ=0.49	Neigh STFT	sym5 Wavelet SURE, Level 6	db13 Wavelet SURE, Level 5	coif1 Wavelet SURE, Level 7
ISNR (db)	11.1639	11.8899	**12.6307**	8.9827	7.3205	5.7691	10.1766	12.1502	9.7551
PSNR (db)	30.3510	31.0770	**31.8178**	28.1813	26.5192	24.9562	29.3636	31.3373	28.9421
RMSE	0.0304	0.0279	**0.0257**	0.0390	0.0472	0.0565	0.0340	0.0271	0.0357
MAE	0.0225	0.0212	0.0195	0.0258	0.0310	0.0377	0.0246	**0.0193**	0.0255
MAX	0.1642	0.1293	**0.1100**	0.2377	0.3011	0.2972	0.3372	0.1719	0.4001

**Table 2:** Denoising results for the CCSN signal s20a1o05 at a distance of 10 kpc (SNR = −2.11 dB). The best performance indices are marked in bold.

Perform. Index	LPA-RICI n=0 Γ=11.25, $R_{c}=1$	LPA-RICI n=1 Γ=13, $R_{c}=1$	LPA-RICI n=2 Γ=24, $R_{c}=1$	LPA-ICI Γ=1.25	TV μ=0.26	Neigh STFT	sym4 Wavelet SURE, Level 6	db13 Wavelet SURE, Level 5	coif4 Wavelet SURE, Level 5
ISNR (db)	13.3754	13.4002	**14.0397**	11.6172	9.4346	10.2554	11.4397	12.6607	10.4296
PSNR (db)	26.5485	26.5733	**27.2127**	24.7953	22.6127	23.4284	24.6127	25.8338	23.6026
RMSE	0.0471	0.0469	**0.0436**	0.0576	0.0740	0.0674	0.0588	0.0511	0.0660
MAE	0.0364	0.0357	**0.0326**	0.0388	0.0439	0.0419	0.0379	0.0369	0.0421
MAX	0.2309	0.2200	**0.2200**	0.4277	0.4876	0.5204	0.7805	0.3323	0.8102

**Table 3:** Denoising results for the CCSN signal s20a1o05 at a distance of 20 kpc (SNR = −8.13 dB). The best performance indices are marked in bold.

Perform. Index	LPA-RICI n=0 Γ=20.5, $R_{c}=1$	LPA-RICI n=1 Γ=20, $R_{c}=1$	LPA-RICI n=2 Γ=28, $R_{c}=1$	LPA-ICI Γ=1.5	TV μ=0.31	Neigh STFT	sym3 Wavelet SURE, Level 7	db3 Wavelet SURE, Level 7	coif1 Wavelet SURE, Level 7
ISNR (db)	15.5200	15.3378	**15.6755**	14.2550	12.5169	13.8119	13.1240	13.1240	11.4983
PSNR (db)	22.6734	22.4912	**22.8289**	21.4124	19.6743	20.9653	20.2774	20.2774	18.6518
RMSE	0.0735	0.0751	**0.0722**	0.0850	0.1038	0.0895	0.0969	0.0969	0.1168
MAE	0.0537	0.0570	0.0551	0.0547	0.0618	**0.0471**	0.0644	0.0644	0.0775
MAX	**0.4227**	0.4399	0.4399	0.6901	0.6482	1.0478	1.4032	1.4032	1.5769

**Table 4:** Relative performance improvement of the LPA-RICI-based ($n=2,\Gamma =11,R_{c}=1$) denoising over other tested methods, for the CCSN signal s20a1o05 at a distance of 5 kpc (SNR = 3.9 dB).

Perform. Index	LPA-ICI Γ=0.75	TV μ=0.49	Neigh STFT	sym5 Wavelet SURE, Level 6	db13 Wavelet SURE, Level 5	coif1 Wavelet SURE, Level 7
ISNR	40.61%	72.54%	118.94%	24.12%	3.95%	29.48%
PSNR	12.90%	19.98%	27.49%	8.36%	1.53%	9.94%
RMSE	34.10%	45.55%	54.51%	24.41%	5.17%	28.01%
MAE	24.42%	37.10%	48.28%	20.73%	−1.04%	23.53%
MAX	53.72%	63.47%	62.99%	67.38%	36.01%	72.51%

**Table 5:** Relative performance improvement of the LPA-RICI-based ($n=2,\Gamma =24,R_{c}=1$) denoising over other tested methods, for the CCSN signal s20a1o05 at a distance of 10 kpc (SNR = −2.11 dB).

Perform. Index	LPA-ICI Γ=1.25	TV μ=0.26	Neigh STFT	sym4 Wavelet SURE, Level 6	db13 Wavelet SURE, Level 5	coif4 Wavelet SURE, Level 5
ISNR	20.85%	48.81%	36.90%	22.73%	10.89%	34.61%
PSNR	9.75%	20.34%	16.15%	10.56%	5.34%	15.30%
RMSE	24.31%	41.08%	35.31%	25.85%	14.68%	33.94%
MAE	15.98%	25.74%	22.20%	13.98%	11.65%	22.57%
MAX	48.56%	54.88%	57.72%	71.81%	33.79%	72.85%

**Table 6:** Relative performance improvement of the LPA-RICI-based ($n=2,\Gamma =28,R_{c}=1$) denoising over other tested methods, for the CCSN signal s20a1o05 at a distance of 20 kpc (SNR = −8.13 dB).

Perform. Index	LPA-ICI Γ=1.5	TV μ=0.31	Neigh STFT	sym3 Wavelet SURE, Level 7	db3 Wavelet SURE, Level 7	coif1 Wavelet SURE, Level 7
ISNR	9.96%	25.23%	13.49%	19.44%	19.44%	36.33%
PSNR	6.62%	16.03%	8.89%	12.58%	12.58%	22.40%
RMSE	15.06%	30.44%	19.33%	25.49%	25.49%	38.18%
MAE	−0.73%	10.84%	−16.99%	14.44%	14.44%	28.90%
MAX	36.26%	32.14%	58.02%	68.65%	68.65%	72.10%

**Table 7:** Algorithm execution times of the tested denoising methods, for the CCSN signal s20a1o05 at distances of 5, 10, and 20 kpc.

Execution Time (s)									
**Distance** **(kpc)**	**LPA-RICI** n=0	**LPA-RICI** n=1	**LPA-RICI** n=2	**LPA-ICI**	**TV**	**Neigh** **STFT**	**Symlet** **Wavelet**	**Daubechies** **Wavelet**	**Coiflet** **Wavelet**
5	0.3245	0.4239	0.7288	3.0619	0.0154	0.7661	0.0061	0.0059	0.0046
10	0.4839	0.5923	1.3046	4.6105	0.0162	0.7967	0.0073	0.0086	0.0055
20	0.8352	0.8296	1.3178	7.3022	0.0160	0.7457	0.0058	0.0059	0.0058

**Table 8:** Denoising results for the CCSN signal s20a2o09 at a distance of 5 kpc (SNR = −4.54 dB). The best performance indices are marked in bold.

Perform. Index	LPA-RICI n=0 Γ=9, $R_{c}=1$	LPA-RICI n=1 Γ=13, $R_{c}=1$	LPA-RICI n=2 Γ=16, $R_{c}=1$	LPA-ICI Γ=1.25	TV μ=0.24	Neigh STFT	sym4 Wavelet SURE, Level 6	db6 Wavelet SURE, Level 6	coif1 Wavelet SURE, Level 7
ISNR (db)	12.1897	11.8699	12.2523	11.0513	**12.4081**	10.4299	11.2266	11.6597	12.2478
PSNR (db)	21.1651	20.8453	21.2277	20.0267	**21.3835**	19.4053	20.2021	20.6351	21.2232
RMSE	0.0596	0.0619	0.0592	0.0680	**0.0582**	0.0730	0.0666	0.0634	0.0592
MAE	0.0443	0.0428	0.0424	0.0416	0.0412	0.0438	0.0470	0.0439	**0.0405**
MAX	0.3201	0.3030	0.2913	0.3627	0.2985	0.5463	0.4402	0.3855	**0.2391**

**Table 9:** Denoising results for the CCSN signal s20a2o09 at a distance of 10 kpc (SNR = −10.09 dB). The best performance indices are marked in bold.

Perform. Index	LPA-RICI n=0 Γ=13.5, $R_{c}=1$	LPA-RICI n=1 Γ=20, $R_{c}=1$	LPA-RICI n=2 Γ=28, $R_{c}=1$	LPA-ICI Γ=1.5	TV μ=0.27	Neigh STFT	sym4 Wavelet SURE, Level 6	db13 Wavelet SURE, Level 5	coif1 Wavelet SURE, Level 7
ISNR (db)	13.7334	13.5924	**14.2997**	13.1394	13.9226	7.8194	11.1812	12.0025	12.5731
PSNR (db)	17.1611	17.0201	**17.7275**	16.5671	17.3504	11.2472	14.6089	15.4303	16.0009
RMSE	0.0946	0.0961	**0.0886**	0.1012	0.0925	0.1868	0.1269	0.1154	0.1081
MAE	0.0704	0.0667	0.0636	**0.0581**	0.0595	0.0802	0.0887	0.0870	0.0735
MAX	0.5082	0.5237	**0.4489**	0.5831	0.6238	2.3191	0.8801	0.6794	0.6437

**Table 10:** Denoising results for the CCSN signal s20a2o09 at a distance of 20 kpc (SNR = −15.98 dB). The best performance indices are marked in bold.

Perform. Index	LPA-RICI n=0 Γ=5, $R_{c}=0.9$	LPA-RICI n=1 Γ=26, $R_{c}=1$	LPA-RICI n=2 Γ=30, $R_{c}=1$	LPA-ICI Γ=1.5	TV μ=0.28	Neigh STFT	sym4 Wavelet SURE, Level 6	db13 Wavelet SURE, Level 5	coif1 Wavelet SURE, Level 7
ISNR (db)	17.2693	15.9216	15.8755	16.1431	**17.3306**	8.8306	13.2998	12.4740	13.8150
PSNR (db)	14.8028	13.4551	13.4090	13.6766	**14.8641**	6.3641	10.8333	10.0075	11.3485
RMSE	0.1241	0.1449	0.1456	0.1412	**0.1232**	0.3277	0.1959	0.2155	0.1846
MAE	**0.0698**	0.1012	0.1084	0.0769	0.0759	0.1072	0.1145	0.1572	0.1059
MAX	**0.6685**	0.9006	0.9006	0.9036	0.7929	4.0053	3.0110	1.3596	2.7521

**Table 11:** Relative performance improvement of the LPA-RICI-based ($n=2,\Gamma =16,R_{c}=1$) denoising over other tested methods, for the CCSN signal s20a2o09 at a distance of 5 kpc (SNR = −4.54 dB).

Perform. Index	LPA-ICI Γ=1.25	TV μ=0.24	Neigh STFT	sym4 Wavelet SURE, Level 6	db6 Wavelet SURE, Level 6	coif1 Wavelet SURE, Level 7
ISNR	10.87%	−1.26%	17.47%	9.14%	5.08%	0.04%
PSNR	6.00%	−0.73%	9.39%	5.08%	2.87%	0.02%
RMSE	12.94%	−1.72%	18.90%	11.11%	6.62%	0.00%
MAE	−1.92%	−2.91%	3.20%	9.79%	3.42%	−4.69%
MAX	19.69%	2.41%	46.68%	33.83%	24.44%	−21.83%

**Table 12:** Relative performance improvement of the LPA-RICI-based ($n=2,\Gamma =28,R_{c}=1$) denoising over other tested methods, for the CCSN signal s20a2o09 at a distance of 10 kpc (SNR = −10.09 dB).

Perform. Index	LPA-ICI Γ=1.5	TV μ=0.27	Neigh STFT	sym4 Wavelet SURE, Level 6	db13 Wavelet SURE, Level 5	coif1 Wavelet SURE, Level 7
ISNR	8.83%	2.71%	82.87%	27.89%	19.14%	13.73%
PSNR	7.00%	2.17%	57.62%	21.35%	14.89%	10.79%
RMSE	12.45%	4.22%	52.57%	30.18%	23.22%	18.04%
MAE	−9.47%	−6.89%	20.70%	28.30%	26.90%	13.47%
MAX	23.01%	28.04%	80.64%	48.99%	33.93%	30.26%

**Table 13:** Relative performance improvement of the LPA-RICI-based ($n=0,\Gamma =5,R_{c}=0.9$) denoising over other tested methods, for the CCSN signal s20a2o09 at a distance of 20 kpc (SNR = −15.98 dB).

Perform. Index	LPA-ICI Γ=1.5	TV μ=0.28	Neigh STFT	sym4 Wavelet SURE, Level 6	db13 Wavelet SURE, Level 5	coif1 Wavelet SURE, Level 7
ISNR	6.98%	−0.35%	95.56%	29.85%	38.44%	25.00%
PSNR	8.23%	−0.41%	132.60%	36.64%	47.92%	30.44%
RMSE	12.11%	−0.73%	62.13%	36.65%	42.41%	32.77%
MAE	9.23%	8.04%	34.89%	39.04%	55.60%	34.09%
MAX	26.02%	15.69%	83.31%	77.80%	50.83%	75.71%

**Table 14:** Algorithm execution times of the tested denoising methods, for the CCSN signal s20a2o09 at distances of 5, 10, and 20 kpc.

Execution Time (s)									
**Distance** **(kpc)**	**LPA-RICI** n=0	**LPA-RICI** n=1	**LPA-RICI** n=2	**LPA-ICI**	**TV**	**Neigh** **STFT**	**Symlet** **Wavelet**	**Daubechies** **Wavelet**	**Coiflet** **Wavelet**
5	0.3427	0.5322	0.6698	4.7678	0.0231	0.7854	0.0054	0.0054	0.0058
10	0.4770	0.7887	1.2294	9.5808	0.0218	0.7553	0.0054	0.0071	0.0059
20	0.1670	1.0763	1.3092	12.6891	0.0231	0.7768	0.0042	0.0071	0.0058

**Table 15:** Denoising results for the CCSN signal s20a3o15 at a distance of 5 kpc (SNR = −2.18 dB). The best performance indices are marked in bold.

Perform. Index	LPA-RICI n=0 Γ=10.75, $R_{c}=1$	LPA-RICI n=1 Γ=16, $R_{c}=1$	LPA-RICI n=2 Γ=20, $R_{c}=1$	LPA-ICI Γ=1	TV μ=0.38	Neigh STFT	sym4 Wavelet SURE, Level 6	db25 Wavelet SURE, Level 4	coif4 Wavelet SURE, Level 5
ISNR (db)	12.9255	**12.9800**	12.8344	10.7381	9.6876	7.2057	10.5605	11.8525	10.0418
PSNR (db)	22.4275	**22.4820**	22.3364	20.2401	19.1895	16.7077	20.0624	21.3545	19.5438
RMSE	0.0756	**0.0751**	0.0764	0.0973	0.1098	0.1461	0.0993	0.0856	0.1054
MAE	0.0590	0.0557	**0.0546**	0.0641	0.0774	0.0877	0.0679	0.0676	0.0671
MAX	**0.3054**	0.4222	0.3502	0.4744	0.4466	0.7650	1.0834	0.3646	1.2784

**Table 16:** Denoising results for the CCSN signal s20a3o15 at a distance of 10 kpc (SNR = −8.17 dB). The best performance indices are marked in bold.

Perform. Index	LPA-RICI n=0 Γ=17.5, $R_{c}=1$	LPA-RICI n=1 Γ=20, $R_{c}=1$	LPA-RICI n=2 Γ=26, $R_{c}=1$	LPA-ICI Γ=1.25	TV μ=0.3	Neigh STFT	sym8 Wavelet SURE, Level 5	db4 Wavelet SURE, Level 6	coif4 Wavelet SURE, Level 5
ISNR (db)	**15.6555**	15.4472	14.9058	12.9291	11.9438	10.3813	11.3717	12.6203	10.7677
PSNR (db)	**19.1655**	18.9571	18.4157	16.4390	15.4537	13.8912	14.8816	16.1303	14.2776
RMSE	**0.1101**	0.1128	0.1200	0.1507	0.1688	0.2020	0.1803	0.1561	0.1932
MAE	**0.0845**	0.0850	0.0890	0.1002	0.1123	0.1223	0.1102	0.1044	0.1194
MAX	**0.6060**	0.6680	0.6680	0.9034	0.6923	1.2354	2.1975	0.9295	2.3395

**Table 17:** Denoising results for the CCSN signal s20a3o15 at a distance of 20 kpc (SNR = −14.19 dB). The best performance indices are marked in bold.

Perform. Index	LPA-RICI n=0 Γ=22, $R_{c}=1$	LPA-RICI n=1 Γ=25, $R_{c}=1$	LPA-RICI n=2 Γ=44, $R_{c}=1$	LPA-ICI Γ=1.5	TV μ=0.29	Neigh STFT	sym5 Wavelet SURE, Level 6	db6 Wavelet SURE, Level 6	coif1 Wavelet SURE, Level 7
ISNR (db)	**18.0346**	17.6215	17.6932	15.4138	15.7267	9.0143	12.0204	12.6513	11.3468
PSNR (db)	**15.5324**	15.1193	15.1910	12.9116	13.2245	6.5121	9.5182	10.1491	8.8446
RMSE	**0.1673**	0.1754	0.1740	0.2262	0.2182	0.4725	0.3343	0.3108	0.3612
MAE	0.1299	0.1335	**0.1284**	0.1415	0.1411	0.2302	0.2236	0.1835	0.2325
MAX	**0.7221**	1.3360	1.3360	1.4224	0.9131	3.1182	3.8350	3.7716	4.7470

**Table 18:** Relative performance improvement of the LPA-RICI-based ($n=1,\Gamma =16,R_{c}=1$) denoising over other tested methods, for the CCSN signal s20a3o15 at a distance of 5 kpc (SNR = −2.18 dB).

Perform. Index	LPA-ICI Γ=1	TV μ=0.38	Neigh STFT	sym4 Wavelet SURE, Level 6	db25 Wavelet SURE, Level 4	coif4 Wavelet SURE, Level 5
ISNR	20.88%	33.99%	80.14%	22.91%	9.51%	29.26%
PSNR	11.08%	17.16%	34.56%	12.06%	5.28%	15.03%
RMSE	22.82%	31.60%	48.60%	24.37%	12.27%	28.75%
MAE	13.10%	28.04%	36.49%	17.97%	17.60%	16.99%
MAX	11.00%	5.46%	44.81%	61.03%	−15.80%	66.97%

**Table 19:** Relative performance improvement of the LPA-RICI-based ($n=0,\Gamma =17.5,R_{c}=1$) denoising over other tested methods, for the CCSN signal s20a3o15 at a distance of 10 kpc (SNR = −8.17 dB).

Perform. Index	LPA-ICI Γ=1.25	TV μ=0.3	Neigh STFT	sym8 Wavelet SURE, Level 5	db4 Wavelet SURE, Level 6	coif4 Wavelet SURE, Level 5
ISNR	21.09%	31.08%	50.80%	37.67%	24.05%	45.39%
PSNR	16.59%	24.02%	37.97%	28.79%	18.82%	34.23%
RMSE	26.94%	34.77%	45.50%	38.94%	29.47%	43.01%
MAE	15.67%	24.76%	30.91%	23.32%	19.06%	29.23%
MAX	32.92%	12.47%	50.95%	72.42%	34.80%	74.10%

**Table 20:** Relative performance improvement of the LPA-RICI-based ($n=0,\Gamma =22,R_{c}=1$) denoising over other tested methods, for the CCSN signal s20a3o15 at a distance of 20 kpc (SNR = −14.19 dB).

Perform. Index	LPA-ICI Γ=1.5	TV μ=0.29	Neigh STFT	sym5 Wavelet SURE, Level 6	db6 Wavelet SURE, Level 6	coif1 Wavelet SURE, Level 7
ISNR	17.00%	14.68%	100.07%	50.03%	42.55%	58.94%
PSNR	20.30%	17.45%	138.52%	63.19%	53.04%	75.61%
RMSE	26.04%	23.33%	64.59%	49.96%	46.17%	53.68%
MAE	8.20%	7.94%	43.57%	41.91%	29.21%	44.13%
MAX	49.23%	20.92%	76.84%	81.17%	80.85%	84.79%

**Table 21:** Algorithm execution times of the tested denoising methods, for the CCSN signal s20a3o15 at distances of 5, 10, and 20 kpc.

Execution Time (s)									
**Distance** **(kpc)**	**LPA-RICI** n=0	**LPA-RICI** n=1	**LPA-RICI** n=2	**LPA-ICI**	**TV**	**Neigh** **STFT**	**Symlet** **Wavelet**	**Daubechies** **Wavelet**	**Coiflet** **Wavelet**
5	0.4498	0.7315	0.9687	3.8876	0.0163	0.7809	0.0054	0.0162	0.0051
10	0.6978	0.8143	1.1634	4.9161	0.0169	0.7755	0.0050	0.0054	0.0050
20	0.8756	1.0480	2.3415	10.5730	0.0169	0.7700	0.0054	0.0055	0.0058

## Abbreviations

The following abbreviations are used in this manuscript:

AR	autoregressive
BBH	binary black holes
BNS	binary neutron star
CBC	compact binary coalescence
CCSN	core collapse supernova
CNN	convolutional neural network
EGO	European Gravitational Observatory
ICI	intersection of confidence intervals
ISI	internal seismic isolation
ISNR	improvement in the signal-to-noise ratio
LIGO	Laser Interferometer Gravitational-Wave Observatory
LPA	local polynomial approximation
MAE	mean absolute error
MAX	maximum absolute error
MSE	mean squared error
NSBH	neutron star-black hole
PCA	principal component analysis
PSNR	peak signal-to-noise ratio
RICI	relative intersection of confidence intervals
RMSE	root mean squared error
ROF	Rudin–Osher–Fatemi
SNR	signal-to-noise ratio
STFT	Short-time Fourier transform
SURE	Stein’s unbiased risk estimator
TV	total variation
WLS	weighted least squares
